# Home telemonitoring in smart rurality: results from the HIS2R interreg feasibility pilot study

**DOI:** 10.1007/s40520-024-02709-7

**Published:** 2024-03-13

**Authors:** Didier Schoevaerdts, Marie-Paule Lerude, Véronique Tellier, Marie Pierard, Dimitri Voilmy, Jean-Luc Novella

**Affiliations:** 1 Department of Geriatric Medicine, CHU UCL Namur, Institute of Health and Society, Catholic University of Louvain, Avenue Dr Gaston Thérasse, 1, 5530 Yvoir, Belgium; 2 Public Health Department, Province de Namur, Place Saint Aubain, 2, 5000 Namur, Belgium; 3 Fédération des Centres de Services à Domicile - FCSD, Rue de Gembloux 196, 5002 Namur, Belgium; 4 Laboratoire Informatique et Société Numérique-Équipe Modélisation et Sûreté des Systèmes, Université de technologie de Troyes, Rue Marie Curie, 12, 10300 Troyes, France; 5 Department of Geriatric Medicine, CHU Reims, Hôpital Maison Blanche, rue Cognacq Jay 45, 51100 Reims, France

**Keywords:** Telemedicine, Case-manager, Heart failure, Chronic obstructive pulmonary disease

## Abstract

**Aims:**

The Health in Smart Rurality Interreg project aims to assess the feasibility of telemonitoring in rural areas across the Franco-Belgian border among patients affected by heart failure or chronic obstructive pulmonary disease. The objectives were to better understand strengths or barriers to implementing telemonitoring for early detection of potential adverse events, for improving quality of life, communication, and care coordination.

**Methods:**

Using a prospective 6-month observational design, interconnected pads were provided to community-dwelling adults aged over 60 years. The device monitored daily body weight, temperature, cardiac rate, blood pressure, and oxygen saturation. Using predefined warning thresholds, data were analyzed by a nurse case-manager who also provided therapeutic education during their contacts.

**Results:**

Out of 87 eligible and screened patients, 21 (24%) were included in the study. At the end of the follow-up, 19 patients (90%) were re-assessed. The rate of hospitalization and mortality was high (32% and 10%, respectively). A total of 644 alerts were recorded (median of 29 alerts/patients) with a high rate of technically-related alerts (TRA) (26%). Out of the 475 non-TRA, 79% and 1% have led to an intervention by the case-manager or the physician, respectively. Therapeutic adjustment was proposed for 12 patients during that period.

**Conclusion:**

Telemonitoring appears to be a promising solution for the follow-up of patients living far from medical resources. The contribution of a case-manager is of added-value in managing alerts, therapeutic education, and coaching. Many questions remain open such as the improvement of technical aspects and long-term compliance in a real-world setting.

## Introduction

Chronic conditions are health problems that place the greatest demands on the healthcare system, in terms of diagnosis, treatment, and decompensation management [1]. Their prevalence increases with age [2]. They often affect frail people at risk of functional decline. The management of these pathologies relies on a hierarchical organization of the healthcare system. In recent years, medical practices have been enriched by the ability to better monitor patients’state of health at home, through the development of telemedicine [3]. This approach provides even greater benefits for populations far from primary or secondary care systems in rural areas. In general, the healthcare system is organized by a country’s health authorities, but there is a specific organization for border areas: Zones Organisées d’Accès aux Soins Transfrontaliers (ZOAST), designed to facilitate the access to healthcare on both sides of the border. The Ardennes ZOAST, created in 2008, is one of seven zones located around the Franco-Belgian border and defined by partners within the Franco-Belgian Health Observatory (OFBS) [4]. From a healthcare perspective, the region is characterized by a low population density over a vast territory, and a low density of healthcare facilities. As a result, access to healthcare can sometimes require considerable traveling. In this context, the organization of care is crucial, and must foster communication and coordination between the actors involved to ensure individualized care adjusted to chronic pathologies, while taking into account people’s frailty level. Heart failure and chronic obstructive pulmonary disease (COPD) are among the most frequent chronic pathologies after the age of 65, with a high risk of exacerbation and hospitalization [5, 6]. Patients suffering from this condition can also benefit from a strategy targeting early detection of warning signs, so that treatment can be adjusted proactively to reduce the severity of critical events. This strategy should make it possible to limit the functional impact of decompensation and the need to resort to a healthcare system that is currently saturated.

This pilot study aims to study the feasibility of setting up a device for recording parameters using an interconnected tablet, supported by a tele-vigilance system and a case-manager in the homes of patients suffering from heart failure or COPD in a cross-border rural area. The aim of this system is to detect and limit the first signs of decompensation, and to immediately adjust the treatment. The aim is also to better coordinate care for frail people living at home in rural areas, to improve the communication between care providers on both sides of the border, and to improve the safety and comfort of life for patients and their families.

## Methods

This is a prospective feasibility study. The study took place in the Ardennes ZOAST area. Patients included in the study had to be over 60 years of age, living at home in a rural environment, suffering from heart failure (NYHA score ≥ 2) or COPD (Gold Stage ≥ 2), and they had to have presented at least one episode of decompensation or exacerbation in the past two years. The study’s exclusion criteria were: not having Internet access, living in a care institution, already having an implantable interconnected device, suffering from severe cognitive impairment (MMSE < 15/30), having poor knowledge of the French or Dutch language, suffering from a terminal illness with a life expectancy estimated at less than 12 months, or being under court protection.

Patients were recruited either by general practitioners or specialists involved in the project, or by study nurses working with hospitalized patients, on the Belgian side at the University Hospital Center (CHU) UCL Namur (Godinne or Dinant sites), at the Centre de Santé des Fagnes in Chimay and, on the French side, at the Centre Hospitalier Intercommunal Nord-Ardennes in Charleville-Mézières.

After signing informed consent, all patients were assessed by a nurse case-manager who collected demographic and clinical information. The main clinical data collected at the time of inclusion were: severity of the main pathology, presence of comorbidities (Charlson Comorbidity Index) [7], list of medicines, autonomy in basic or instrumental activities of daily living (24-point Katz activities of daily living [ADL] scale and 8-point Lawton instrumental ADL [iADL] scale) [8, 9], level of frailty (26-point SEGA tool, where a score of ≤ 8/26 indicates the absence of frailty) [10], level of physical performance (12-point Short Physical Performance Battery (SPPB) scale), where a score of ≤ 10/12 indicates poor or intermediate physical performance) [11], nutritional status (14-point Mini Nutritional Assessment-Short Form), and mood (4-point Mini-Geriatric Depression Scale) [12, 13]. Quality of life was assessed using the Medical Outcomes Study Short-Form 12 (SF-12) questionnaire, which generates a simplified physical and mental score from the original SF-36 form [14].

At the end of this first visit, an interconnected tablet (Intersysto company; 3S-Homecare ecosystem) was installed and used to securely collect patient parameters on a daily basis: weight, temperature, heart rate, blood pressure, and oxygen saturation. The data were securely hosted in the Microsoft Azure Cloud, certified under Health Data Hosting regulations. The parameters were then available in real time to the care providers involved in the project. In addition to monitoring, the tool also enabled communication between care providers and the patient, providing a diary of homecare providers, a reminder of tasks to be carried out by the patient, and a link to the Télépronam tele-vigilance system managed by the Province of Namur.

Medical data were securely shared to the patient’s care providers, identified through the Réseau Santé Wallon under the responsibility of the Fédération Régionale des Associations de Télématique Médicale (FRATEM asbl.).

All collected data were encoded in a protected pseudonymized database, using CleanWeb software, and centralized in the methodology unit of the University of Reims Champagne-Ardenne (URCA).

During monitoring, the connected tool identified alerts graded according to four severity levels. These were sent by text messages to the case-manager during the day from Monday to Friday, or to the Télépronam operator in the evening, at night or on weekends. The Telepronam operator had a decision-making algorithm at his disposal, enabling him to standardize his intervention according to the territory in which the patient resided. Alerts generated triggered action by the case-manager. The predefined alert thresholds were weight change of more than 5% or 2 kg in 2 days, temperature ≥ 38°, heart rate < 50/min or 100/minute, systolic blood pressure ≥ 160 mmHg or diastolic blood pressure ≤ 85 mmHg, and oxygen saturation varying by at least 4% between two intakes or < 90%. Based on the level of the alert threshold, the response provided by the case-manager (or the Telepronam operator outside working hours) varied increasingly according to the risk: Grade 1: simple call of the patient by the case-manager; Grade 2: call of the caregiver; Grade 3: call of the general practitioner; Grade 4: direct call of the country’s emergency centre and ambulance dispatch.

Patients were monitored by the case-manager over a 6-month period. At the end of the follow-up period, a final evaluation visit was carried out. During follow-up, patients also benefited from a therapeutic education program and motivational talks provided by the case-manager, who had undergone a specific training program.

The following critical events were recorded: the need to consult one’s general practitioner or specialist, recourse to therapeutic adjustment, adaptation of home aids, the occurrence of a fall, hospitalization, and eventually death.

A qualitative study was carried out at the end of the follow-up period, using a qualitative questionnaire on the usability of the system and a semi-structured interview with the participants, to identify the obstacles and drivers to the implementation of this organization.

Descriptive statistical analyses were performed using SAS software. Categorical data are presented as absolute numbers and percentages. Continuous variables are expressed as means and standard deviations. Comparisons between data at inclusion and at the end of the follow-up period were made using the Chi-square test for categorical variables and the paired Student’s t test for continuous data.

In accordance with the Law of May 7, 2004, the study has received the approval of the main Medical Ethics Committee of CHU UCL Namur-site of Godinne (NUB: B0392020000013-internal number 53/2020) and the secondary Ethics Committees (CHU UCL Namur site of Dinant and Centre de Santé des Fagnes, Chimay). It was also approved by all the Data Protection Officers (DPOs) of the different partners in accordance with the General Data Protection Regulation (GDPR) of May 2018. The study has also received a favorable opinion from the Comité de Protection des Personnes Sud-Est 1 (2020-119) and the Commission Nationale de l’Informatique et des Libertés (CNIL) in France.

## Results

During the inclusion period (from July 1, 2020 to June 30, 2021), 87 patients were identified as eligible. Of these, 5 could not be reached and 54 refused to take part in the study. Of the 28 who initially agreed to take part in the project, 7 refused to have the device installed. Overall, 21 patients were therefore included in the study. Most patients were recruited by research associates from the project’s partner hospitals (*n* = 18). The remaining patients were recruited by a specialist physician (*n* = 2) and a cross-border general practitioner (*n* = 1). Of the 21 patients fitted with the equipment, 14 were Belgians undergoing treatment in Belgium, 5 were French undergoing treatment in Belgium, and 2 were French undergoing treatment in France. A total of ten patients were suffering from heart failure, of which eight were at NYHA Stage 3 or 4. In addition, 15 patients suffered from COPD, 12 of whom had Gold Stage 3 or 4. Of these, four patients had both COPD and heart failure.

Table 1 shows the main characteristics of the patients included in the study.

**Table 1:** Clinical characteristics of included patients in the HIS2R feasibility pilot study (*n* = 21)

Characteristics	
Mean age, years	68.0 ± 5.0
Female gender	11 (52)
Previous occupation	
Manual worker	11 (55)
Employee	6 (30)
Intermediate or higher intellectual profession (executive)	2 (10)
Unemployed	1 (5)
Residency	
House	18 (86)
Apartment	3 (14)
Lifestyle	
With spouse	11 (52)
With parents	4 (19)
Alone	6 (29)
Home assistance	
Informal (family and friends)	12 (71)
Formal	
Housekeeper or family helper	12 (71)
Nurse	4 (36)
Physiotherapy at home	9 (43)
Home-delivered meals	3 (27)
Lifestyle	
Confined to home	6 (30)
Walks near the house	6 (30)
Walks far from the house	8 (40)
Active in community life	19 (85)
Active smoker	7 (37)
Former smoker	12 (63)
Functional status	
ADL score (/24)	5.6 ± 0.7
iADL score (/8)	6.2 ± 1.6
SPPB score ≤ 10/12	12 (57)
Nutritionnal status	
MNA-SF ≤ 11/14	4 (19)
Body Mass Index (kg/m^2^)	31 ± 10
Comorbidities	
Charlson Comorbidity Index	5.1 ± 2.5
Previous myocardial infarction	2 (10)
Congestive heart failure	11 (55)
Peripheral vascular disease	8 (40)
Previous cerebrovascular accident	4 (20)
Diabetes	6 (30)
Duodenal ulcer disease	3 (15)
Moderate or severe kidney disease	5 (25)
Cancer, lymphoma or multiple myeloma	9 (45)
Frailty score, SEGA ≤ 8/26	17 (85)
Possible depression score, mini-GDS ≥ 1/4	13 (65)
Oxygen at home	7 (33)
CPAP nocturnal ventilation	2 (10)
Vaccination status at inclusion	
Influenza	11 (55)
Pneumococcus	13 (65)
Covid	19 (95)
SF-12 score, physical assessment	35.9 ± 9.5
SF-12 score, mental assessment	47.5 ± 6.8

Data are presented in absolute number and their percentage (%) for categorical data and mean ± standard deviation (SD) for continuous data

ADL, Activities of Daily Living (Katz scale /24); HIS2R, Health in Smart Rurality; iADL, instrumental Activities of Daily Living (Lawton scale /8); SF-12, 12-Item Short Form Survey; SEGA, Short Emergency Geriatric Assessment tool; SPPB, Short Physical Performance Battery; MNA-SF, Mini-Nutritionnal Assessment-Short Form; Mini-GDS, Mini-Geriatric Depression Scale; CPAP, continuous positive airway pressure

The average follow-up duration was 5.5 months and two patients died during this period. The first patient died soon after inclusion and the second during follow-up. We therefore collected data at the study end for the 19 surviving patients.

At the end of the 6-month follow-up period, no patient had wished to leave the study. There were no adverse changes in comorbidities, functional status, physical performance, frailty, risk of depression, or physical or mental quality of life (Table 2).

**Table 2:** Evolution of clinical status at the end of the 6-month follow-up period (*n* = 19)

	Inclusion	End of follow-up (6 months)	*P-*value
Charlson Comorbidity Index	5.0 ± 2.5	5.0 ± 2.5	0.330
Functional status			
ADL (/24)	5.6 ± 0.7	5.7 ± 0.7	0.130
iADL (/8)	6.2 ± 1.6	6.2 ± 1.7	0.330
Quality of life, SF-12			
Physical	35.9 ± 9.5	32.6 ± 10.8	0.070
Mental	47.5 ± 6.8	48.7 ± 6.6	0.780
Physical performance, SPPB (/12)			
0–6, low	8 (40)	8 (42)	0.800
7–9, intermediate	8 (40)	8 (42)	
10–12, high	4 (20)	3 (16)	
Frailty, SEGA			
≤ 8/26 (non fragile)	17 (85)	17 (90)	0.900
> 8 and < 11/26 (Fragile)	1 (5)	1 (5)	
≥ 11/26 (very fragile)	2 (10)	1 (5)	
Malnutrition, MNA-SF			1.00
≤ 11/14, at risk	4 (20)	4 (21)	
> 11/14, not at risk	16 (80)	15 (79)	
Depression risk, mini-GDS			0.650
≥ 1/4, at risk	13 (65)	11 (58)	
0/4, not at risk	7 (35)	8 (42)	

ADL, Activities of Daily Living (Katz scale /24); iADL, instrumental Activities of Daily Living (Lawton scale /8); SF-12, 12-Item Short Form Survey; SPPB, Short Physical Performance Battery; SEGA, Short Emergency Geriatric Assessment Tool; MNA-SF, Mini-Nutritionnal Assessment-Short Form; Mini-GDS, Mini-Geriatric Depression Scale

Overall, 644 alerts were recorded, with a median number of 29 (ranging from four to 84 alerts). Details of the alerts are shown in Table 3. A high rate of technically-related alerts (TRA) was recorded (*n* = 169; 26%) and included manipulation errors, duplicates, display errors and measuring device dysfunction.

**Table 3:** Characteristics of alerts during the follow-up period

	*N*	%
Technically-related alerts	169	26
Manipulation error	101	60
Duplicate	26	15
Display error	23	14
Measuring device dysfunction	19	11
Non technically-related alerts	475	74
Blood pressure	165	35
Oxygen saturation	118	25
Weigth	116	24
Heart rate	76	16
Temperature	2	< 1
Level of alerts		
Level 1	375	
Level 2	93	
Level 3	6	
Level 4	1	

The main problems leading to non technically-related alerts (non-TRA) were either related to a blood pressure problem (*n* = 165 [35%]), a saturation problem (*n* = 118 [25%]), a weight-related problem (*n* = 116 [24%]), a heart-rate problem (*n* = 76 [16%]), or a temperature problem (*n* = 2 [0.4%]). Of these alerts, 18 concerned 12 different patients and led to a consultation with the general practitioner, and one patient consulted his referring specialist twice.

A total of 20 therapeutic adjustments were made to 12 different patients following the alerts. These adjustments consisted mainly in modifying the hypotensive medication, diuretic doses, and dietary advice following nutritional problems. In two cases, the alerts led to the adaptation of formal home help. During the alerts, eight hospitalizations were recorded in three different patients. At follow-up, two falls were reported during the alerts.

The qualitative part of the study revealed the following main points for the 19 monitored patients: the tablet was an easy-to-use device for the majority (agreement: *n* = 19); the tablet enabled them to better understand their symptoms (agreement: *n* = 12); the tablet enabled the patient to be involved in their monitoring (agreement: *n* = 14); the tablet enabled the patient to be reassured (agreement: *n* = 10); the tablet helped to improve the patient’s well-being (agreement: *n* = 10). The elements that had little impact were the following: the tablet had little impact on the frequency of provider visits (agreement: *n* = 14); the tablet had little impact on the involvement of caregivers (agreement: *n* = 11); the tablet enabled time saving (agreement: *n* = 9).

## Discussion

This pilot study assessed the feasibility of setting up a system combining telemonitoring supported by a telemonitoring system and case management in a rural setting for elderly homecare patients suffering from heart failure and/or COPD in the Ardennes Franco-Belgian cross-border ZOAST region. The aim of this study was not to evaluate the effectiveness of a telemedicine system as this issue has already been addressed in various meta-analyses [15–17]. In our study, the proposed device seems to be adapted to the profile of selected patients whose autonomy is rather preserved. Despite this selection, the system enabled us to identify a relatively large number of early legitimate alarms and therapeutic adjustments. From this point of view, our results are in line with two recent meta-analyses that show a reduction in hospitalizations linked to telemonitoring in heart failure and COPD [18, 19]. In addition, our patients had a fairly high level of comorbidities (Charlson Comorbidity Index 5) and geriatric syndromes (depression, frailty, sarcopenia), which justify a coupled organization between monitoring and case-manager support for therapeutic education and motivational interviewing. Thus, the combination between technology and human resources to help people deal with health problems at home is a helpful way to cover a wider range of needs than those linked only to the pathology, by taking a more global approach to people.

The system has enabled better coordination of care for frail people at home, via a personalized human factor combining good collaboration between the case-manager and front-line providers in the event of an alert. This association between technology and personalized support will undoubtedly allow us to better address the needs of complex patients whose paths are less easily predictable, and who require better care coordination [19]. Telemedicine thus reinforces the case-manager’s action, who can add other intervention methods to face-to-face interviews and telephone contacts [20].

After being equipped, no patient gave up. Patient satisfaction was overall positive and provided a sense of security for them and their family and friends. It has led to a better understanding of the disease and, consequently, to greater involvement, generating more appropriate behaviors. Patients were also more motivated. Here too, the case-manager was seen as a trusted confidant, helping to overcome isolation. Her role enabled sustained, interactive, and educational human guidance throughout the project. A systematic review published in 2017 also identified factors that could improve patient satisfaction and hence telemedicine adherence. These factors were to offer low-cost, easy-to-use modalities, improved clinical outcomes, improved communication, and reduced traveling [21].

We also observed the need to switch from a medical-centric approach, where the patient abides to a system, to a more patient-centric approach, where the system can be optimized for the patient. This means personalizing the entire process (the level of parameters and alerts, therapeutic education, management of comorbidities, etc.).

This type of system often raises the question of how to manage alerts and which professionals need to intervene. One of the strengths of this model, which combines telemonitoring and the case-manager, is its ability to provide an appropriate response to the patient by a first-level responder (the case-manager) who knows the patient well, without overloading general practitioners or specialists. Over a 6-month period, the system-identified situations requiring therapeutic adjustments for over 80% of patients. These adjustments mainly involved adapting hypotensive and diuretic medications, as well as dietary advice.

The assessment of the impact of the system on quality of life was more varied. The qualitative part suggested improved mental well-being. A meta-analysis published in 2017 showed that telemedicine can improve the overall quality of life of patients treated for heart failure [22].

The weaknesses of the study are as follows:

Patient inclusion took place during the Covid-19 pandemic and lasted 6 months less than initially planned. Recruitment was interrupted as a result of health measures, and the context was undoubtedly responsible for some of the refusals to participate in the project. As a result, the number of subjects included was lower than initially planned and we present here results of preliminary data. However, some important key messages derived from that pilot project are underlined.

Measurement sensors undeniably provided useful information that were able to trigger alerts. Patients and caregivers could monitor the trends in vital parameters and this enabled patients to take more control of their health. On the other hand, we have identified that at least a quarter of alerts were false, and therefore this issue needs to be improved by a reliable system that can be used on a larger scale. Some patients could not connect for technical reasons, such as lack of Internet access (white zones near borders), or for reasons related to the digital divide. Like other authors, we believe that we need to be particularly careful so as not to increase social disparities in health in particularly at-risk populations, by improving their level of technical, digital, and health education [23].

Our project shows that ZOASTs are interesting tools for facilitating cross-border care. They enable patients to move easily between hospitals, receive care, or even enter a rehabilitation process. One of the difficulties identified was the need for a shared cross-border medical record, supplied with telemonitoring data and accessible to healthcare professionals on both sides of the border. The computerized contact book was barely used by the front-line medical staff at a time when they were very much in demand due to the health crisis. A future project should therefore include better support for caregivers. The transfer of medical information across the border remained a difficult element in the project, relying more on human-to-human transmission than on technical solutions. According to the 2014 European regulation on electronic identification, the eIDAS program aims to securely share digital data, and is currently being implemented [24]. At the European level, the development of online health information is currently underway in a number of countries (Service européen de santé en ligne [SESALI] system) and could address this issue [25].

If our project were to be generalized, it would have to incorporate more individualized alert targets. From a healthcare efficiency perspective, this type of system would appear to be well-suited to the management of pathologies in rural areas, where access to medical resources is less accessible. Our system could integrate the management of various geriatric syndromes, including the integrated care for older people (iCOPE) approach promoted by the World Health organisation (WHO), which targets self-screening for sensory, locomotor, psychic, cognitive, and nutritional problems as part of the “ageing well” strategy [26]. This type of global approach could be complementary to more targeted approaches to chronic pathologic conditions in elderly populations. It should also enable a more appropriate and coordinated use of the various medical care lines (general practitioners, specialists and hospital practitioners), considering the specificities linked to cross-border links. The impact of such an extended approach combining telemonitoring and case management should be evaluated in a specific randomized controlled study comparing conventional management with an isolated-telemonitoring approach and a third group with telemonitoring and case management.

## Conclusion

Our study therefore highlights the importance of combining technology with a human factor, through the important role played by the case-manager, who ensured optimal use of the technology in the patient’s home, as well as therapeutic education and the transmission of information between the patient’s care providers. This type of approach has proved possible in the management of frail patients, particularly when a border complicates the monitoring and transfer of medical information, thanks to human rather than technological components. It is particularly useful in rural areas, where access to healthcare is more distant from the patient, increasing the accuracy of follow-up for frail patients without requiring them to travel, and giving them the opportunity to participate more actively in their care and understanding of their illness.

## Data availability statement

The data supporting the results presented in the paper can be found by contacting the corresponding author on reasonable request.

## Data Availability

The data supporting the results presented in the paper can be found by contacting the corresponding author on reasonable request.

## Declarations

### Statement of human and animal rigths

All procedures were approved by the primary Medical Ethics Committee of CHU UCL Namur-site of Godinne, the secondary Ethics Committees (CHU UCL Namur site of Dinant and Centre de Santé des Fagnes, Chimay) and Comité de Protection des Personnes Sud-Est 1 (2020-119) and the Commission Nationale de l'Informatique et des Libertés (CNIL) in France.
